# Glial alterations in human prion diseases: A correlative study of astroglia, reactive microglia, protein deposition, and neuropathological lesions

**DOI:** 10.1097/MD.0000000000010320

**Published:** 2018-04-13

**Authors:** Marta Monzón, Rodrigo S. Hernández, Moisés Garcés, Rocío Sarasa, Juan J. Badiola

**Affiliations:** Research Centre for Encephalopathies and Transmissible Emerging Diseases, Institute for Health Research Aragón (IIS), University of Zaragoza, Zaragoza, Spain.

**Keywords:** astrocytes, cerebellum, immunohistochemistry, microglia, neuroinflammation, prion diseases

## Abstract

**Background:**

Neuroinflammation has recently been proposed to be a major component of neurodegenerative diseases. The aim of this study was to determine how the interaction between microglia and astroglia, which are the primary immune cell populations in the brain, and pathological prion protein (PrPsc) could influence the development and propagation of this neurodegenerative disease. Because a relevant role for glial response in prion disease has been clearly demonstrated in our previous studies using the natural animal model, a similar approach has been taken here using the natural human model.

**Methods:**

A morphological approach has been developed to analyze cerebellar samples from patients with Creutzfeldt-Jakob disease (CJD) in comparison with healthy control cases. Histopathological lesions were assessed, and PrPsc, glial fibrillary acidic protein (GFAP) and reactive microglia were immunolabelled by specific antibodies. Furthermore, co-location studies using confocal microscopy were performed to determine the possible relationships between both types of glial cells in all samples.

**Results:**

The results presented in this study support the involvement of both types of glial cells in CJD. Evidence of increased astrocyte and microglia reactivity can be observed in all CJD cases, and a close relationship between the types of glia is demonstrated by co-location studies.

**Conclusion:**

Proteinopathies such as Alzheimer, Parkinson, and Huntington diseases, where aberrant proteins spread throughout the brain during disease progression, may share a molecular basis and mechanisms of propagation. Therefore, studies elucidating the interaction between gliosis and prion propagation may be relevant to these other neurodegenerative diseases and may provide new targets for therapeutic intervention.

## 1 Introduction

According to the current neuroinflammation hypothesis associated with neurodegenerative diseases, the key element underlying the pathogenesis of these disorders involves microglial cells. Their activation is widely assumed to be a marker of pathogenic neuroinflammation in these disorders. Although microglia are generally considered the resident immune cells in the brain, the impact of astrocytes on inflammation cannot be understated.^[1]^ Astrocytes can sense and amplify inflammatory signals from microglia and initiate a series of inflammatory responses, directly affecting neuronal functions. The term gliosis has been applied so broadly that it can either cease to impart useful information or be entirely misleading,^[2]^ starting with inconsistent premises on some occasions. The lack of understanding of the actual neurodegenerative processes likely slows the progress of research in this field. In addition, while the pathogenesis of these neurodegenerative diseases remains unknown, no truly effective treatments can be identified that might prevent the onset or delay the progression of these diseases.

Current evidence supports the idea that many neurodegenerative diseases (those belonging to a prion-like disorder group)^[3]^ involve aberrant protein accumulation, similar to that observed in prion diseases, turning studies elucidating the mechanisms of prion propagation into useful tools for the study of other neurodegenerative diseases.

Although gliosis is always cited as one of the hallmarks of prion diseases, little research has been performed that focused on the involvement of glial cells in prion diseases to elucidate their roles. According to our initial studies regarding the pathogenic progress of Scrapie, as a prototype of prion diseases, astroglia is involved in Scrapie evolution.^[4]^ This glial population has been suggested to sustain active prion propagation^[5]^ and to exist in a close relationship with the vacuoles that form the primary neurodegenerative lesion found in this group of diseases.^[6]^ Based on these encouraging results, the proposal here consists of extending the study on glial involvement from animal to human samples.

Previous studies have focused on the cerebellum for the following reasons. First, based on anatomical and physiological characterization, the cerebellum is particularly vulnerable to insults, and abnormalities in the cerebellum are usually easy to recognize.^[7]^ Second, the cerebellum is one of the areas most affected by pathological prion protein (PrPsc) accumulation, regardless of the subtype associated with the affected individuals, and ataxia is one of the most frequent symptoms. Third, previous studies that focused on microglial cells in prion diseases found no differences between the cerebellum and other brain areas;^[8]^ consequently, the conclusions drawn from studying the cerebellum can be extrapolated to the whole encephalon. In addition, this encephalic area has been proposed to serve as a pseudo-reference region to detect neuroinflammation.^[9]^

The specific goal of this study was to assess whether the expression of astroglia and reactive microglia markers in cerebellar sections from patients with Creutzfeldt–Jakob disease (CJD) differed from those of control patients. This study also aimed to establish a possible relationship between the differential expression of glial and PrPsc deposition and/or neuropathological alterations in human cerebellar samples.

## 2 Materials and methods

### 2.1 Samples

All samples were provided by the human tissue bank from Hospital de Alcorcón (HUFA Biobank, Madrid, Spain), following the guidelines provided by Spanish legislation.

Samples corresponded to a total of 25 CJD patients (age range: 48–82 years; sex: male and female; genotypes at codon 129 of *PRNP*: homozygous and heterozygous), including 22 sporadic and 3 familial cases (*PRNP* E200K mutation). Five additional samples were used as healthy controls (age range: 36–82 years).

Fixed sections (4% paraformaldehyde), corresponding to cerebellar sagittal samples at the vermal level, were obtained and included the granular, Purkinje cell, and molecular layers and white matter for all samples.

The studies performed on samples were approved by the Ethical Committee for Clinical Research from Government of Aragón (CEICA; REFERENCE NUMBER: PI15/0036, Acta No 05/2015).

### 2.2 Methodology

Five-millimeter slices from each individual were paraffin-embedded, from which 5 μm sections were taken and histologically (hematoxylin-eosin staining [HE]) or histochemically processed, following formic acid treatment for prion inactivation. The remaining portions were processed for confocal microscope studies.

To detect PrPsc, GFAP, and reactive microglia, immunohistochemical (IHC) protocols were followed, using specific antibodies in DAKO antibody diluent (Faramount DAKO, Glostrup, Denmark), on sections transferred to Vectabond-treated slides and incubated at 56°C overnight. DAB PLUS was used as a chromogen, and hematoxylin counterstaining and mounting in mounting medium (Faramount DAKO, Glostrup, Denmark) were performed on all sections, regardless of the immunohistochemical protocol applied.

**Figure 1:**
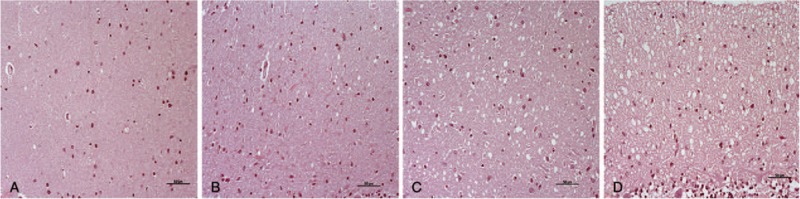
The scoring system used after HE staining to assess spongiosis by light microscopy, according to the number of vacuoles: from (A) very scarce to (D) very abundant (++++). Scale bars: 50 μm. HE = hematoxylin-eosin staining.

**Figure 2:**
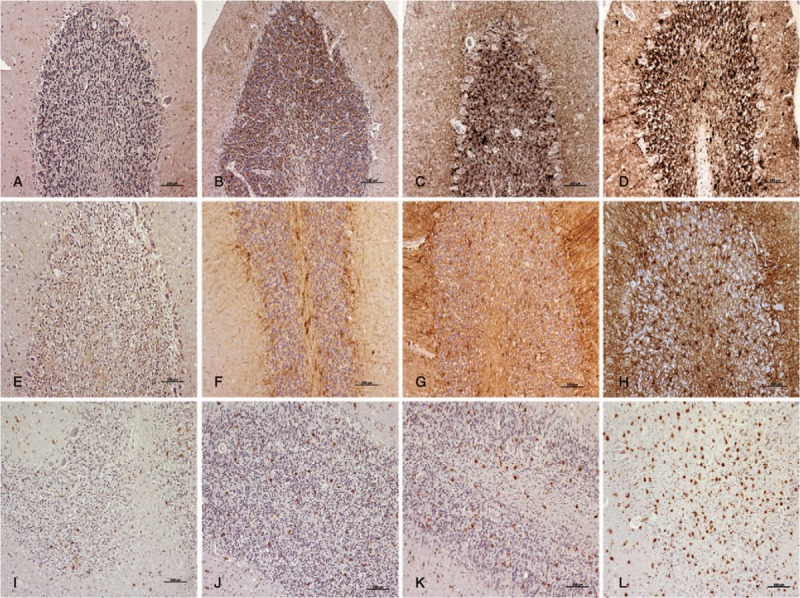
The scoring system used after IHC protocols to evaluate the density and the extent of the labelled deposits (brown; hematoxylin counterstaining, blue) by light microscopy: from minimum to maximum, for PrPsc (A–D); GFAP (E–H); and reactive microglia (I–L). Scale bars: 100 μm. GFAP = glial fibrillary acidic protein, IHC = immunohistochemistry, PrPsc = pathological prion protein.

**Figure 3:**
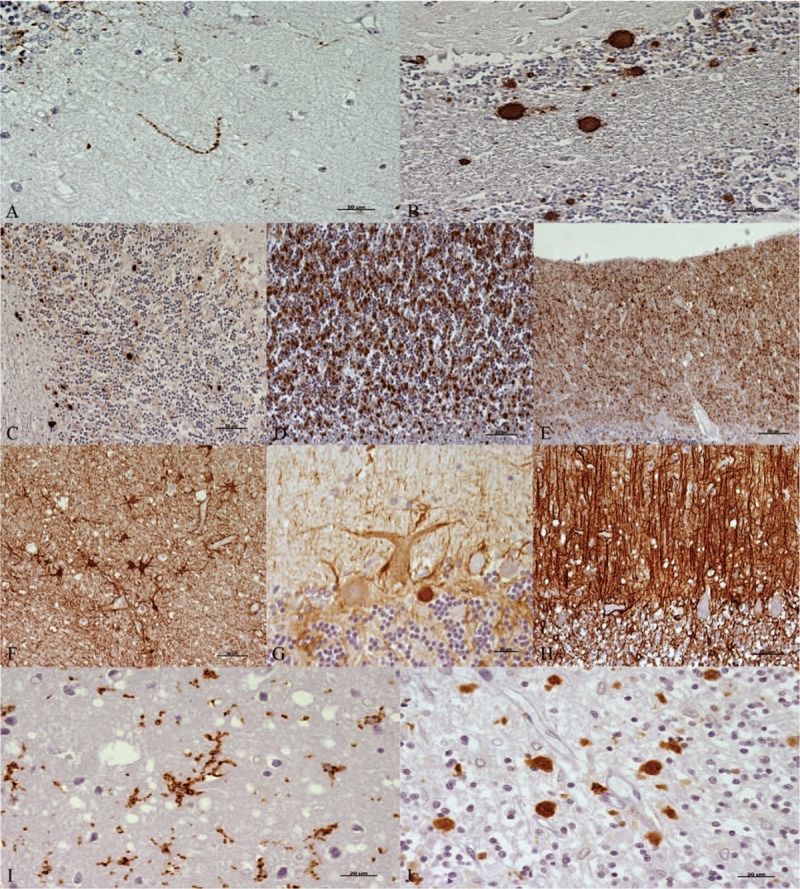
Immunohistochemical patterns observed when each marker (brown; hematoxylin counterstaining, blue) was assessed. For PrPsc: (A) linear, (B) plaques, (C) spots, (D) coalescent, and (E) granular. Scale bars: 20 μm in (A); 50 μm in (B–E). For GFAP: (F) stellate, (G) perineuronal, and (H) radial. Scale bars: 50 μm in F and H; 20 μm in G. For reactive microglia: (I) ramified and (J) amoeboid. Scale bars: 20 μm. GFAP = glial fibrillary acidic protein, PrPsc = pathological prion protein.

After HE staining, all samples were assessed by light microscopy to determine histopathological lesions. All of the layers from each sample were examined for spongiosis and subjectively scored according to the number of vacuoles, that is, (−), absent to (++++), very abundant (Fig. 1). After IHC processing, all marker immunostaining was scored from negative (−) to maximum (++++), by evaluating the density and the extent of the labelled deposits in each layer from each cerebellum, using light microscopy (Fig. 2). The immunostaining morphologies of each marker were also assessed: linear, granular, spot, coalescent or plaques, for PrPsc; stellate, perineuronal or radial, for GFAP; ramified or amoeboid, for reactive microglia (Fig. 3).

Studies using confocal microscopy were performed to assess the possible relationship between astrocytes and reactive microglia in all cases.

#### 2.2.1 Immunohistochemical protocol for PrPsc detection by optical microscopy

It was necessary to include an epitope unmasking protocol, consisting of 96% formic acid immersion (5 minutes), digestion with proteinase K (5 minutes, room temperature [RT]; Life Technologies) and autoclaving in distilled water (10 minutes). After incubation with a primary antibody that recognizes prion protein (12F10 at 1/1700 dilution; SPI Bio, Darmstadt, Germany) for 1 hour at RT, sections were incubated for 30 minutes at RT with EnVision mouse polymer (DAKO, Glostrup, Denmark), which was used as a visualization system.

#### 2.2.2 Immunohistochemical protocol for GFAP detection by optical microscopy

After endogenous peroxidase blocking (H_2_O_2_ 33%) for 5 minutes, sections were incubated with a primary antibody that specifically recognizes glial fibrillary acidic protein (GFAP 1/500, 30 minutes, RT; DAKO), as an astrocytic protein marker. Incubation with the labelled EnVision rabbit polymer (30 minutes, RT; DAKO) was performed.

#### 2.2.3 Immunohistochemical protocol for reactive microglia detection by optical microscopy

Endogenous peroxidase blocking (H_2_O_2_ 33%, 5 minutes), followed by incubation for 30 minutes at RT with major histocompatibility complex type II (MHC II) (1/200; DAKO, Denmark) and CD68 (1/500; DAKO) primary antibodies, was used to immunolabel reactive microglial cells. EnVisionTM polymer (mouse, 30 minutes, RT; DAKO) was used as a visualization system.

#### 2.2.4 Immunohistochemical protocol for confocal microscopy studies

The protocol applied for co-location studies was based on a previously described version.^[5]^ Briefly, 50 μm sections from fixed samples were collected in PBS (floating). Endogenous peroxidase blocking (H2O2 33%, 30 minutes) was followed by incubation with Triton X 100 (0.1% in PBS for 3 hours, RT). Subsequently, incubation with the same specific antibodies, at the same concentrations as described above, was used to individually label reactive microglia and astrocytes (i.e., GFAP, MHC II, and CD68, in PBS-Triton 0.1%). The secondary conjugated antibodies Alexa 488 (1/200; Invitrogen, Eugene, OR) and Alexa 594 (1/200, Invitrogen) were added in PBS for 1 hour in the dark prior to assessment by confocal microscopy (Zeiss LSM 510).

The fluorescence emissions, with excitation from 488 and 594 nm lasers, were detected with a 2-channel multitrack configuration, using bandpass 505 to 530 nm and long-pass 615-nm filters, respectively. Z-stacks of digital images were captured using Zen 2008 software (Carl Zeiss Microimaging, Jena, Germany) and collapsed into 1 resulting picture using the maximum intensity projection provided by the software.

## 3 Results

All samples corresponding to CJD patients showed spongiosis. Spongiosis intensity varied among samples, but it was similar in all layers within each cerebellum, except for 2 occasions. In 1 case, the highest intensity was found in the Purkinje cell layer and the lowest in the granular layer, and the opposite scores were found in the remaining case. Control samples did not show spongiosis in any cases (Table 1 ).

**Table 1:**
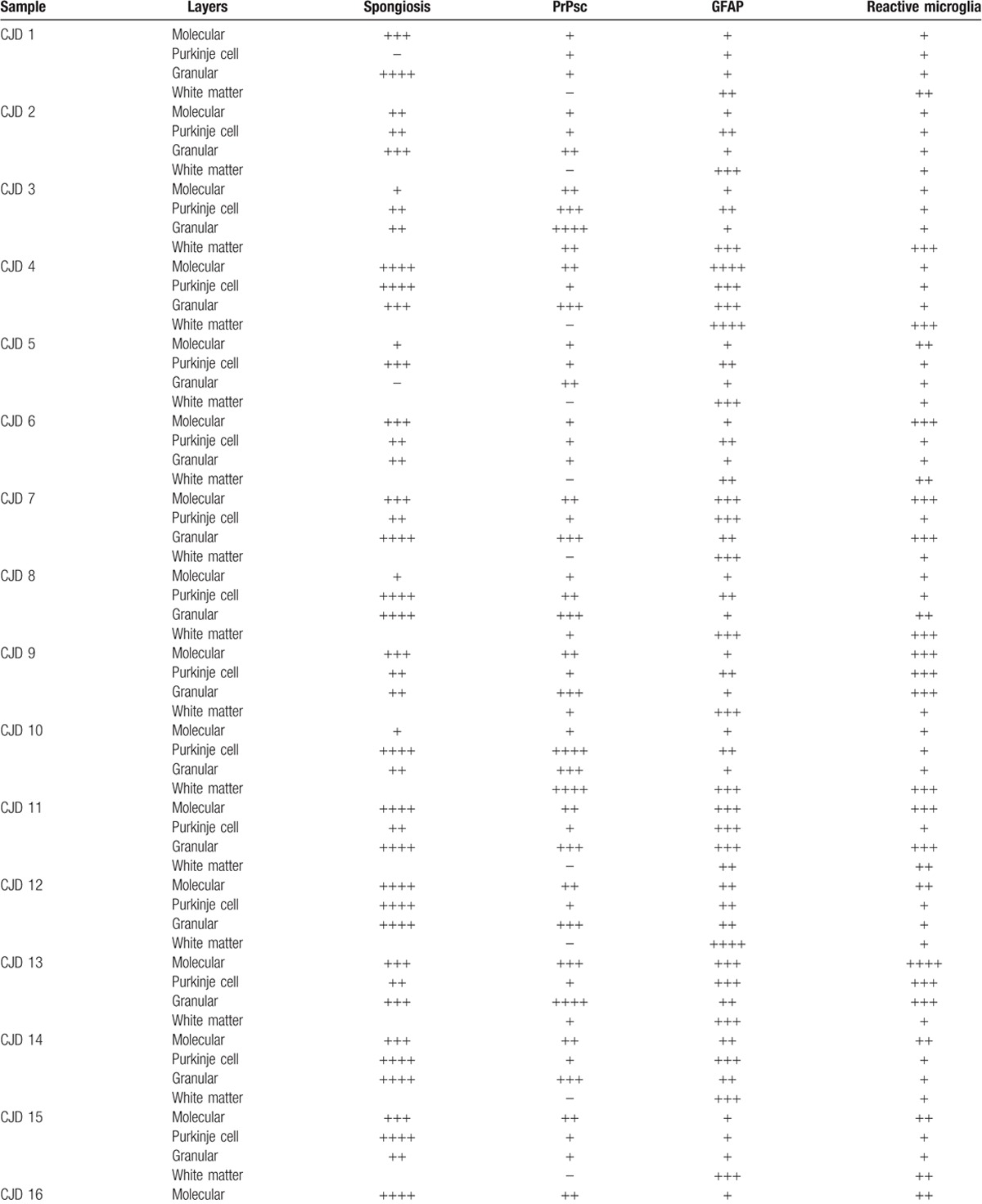
Scores provided by light microscopy examination corresponding to each layer from each cerebellar sample for spongiosis after HE staining (according to the number of vacuoles: −, absence / ++++, very high number in gray matter) or immunolabeling for each specific marker used after IHC processing (by evaluating the density and the extension of the labelling deposits: −, negative / ++++, maximum).

**Table 1 (Continued):**
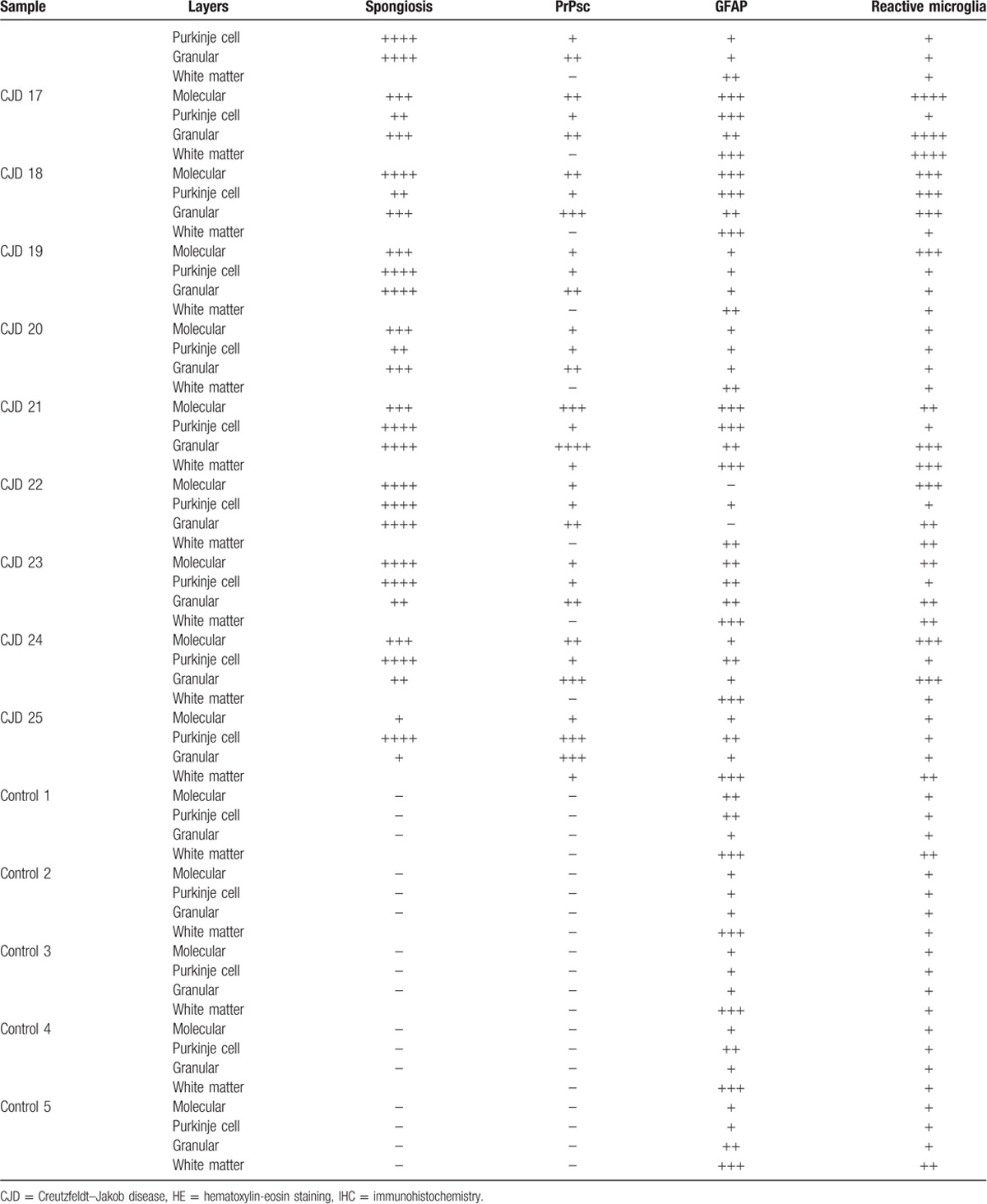
Scores provided by light microscopy examination corresponding to each layer from each cerebellar sample for spongiosis after HE staining (according to the number of vacuoles: −, absence / ++++, very high number in gray matter) or immunolabeling for each specific marker used after IHC processing (by evaluating the density and the extension of the labelling deposits: −, negative / ++++, maximum).

One control and 3 CJD cases presented with the total degeneration of the granular layer.

**Figure 4:**
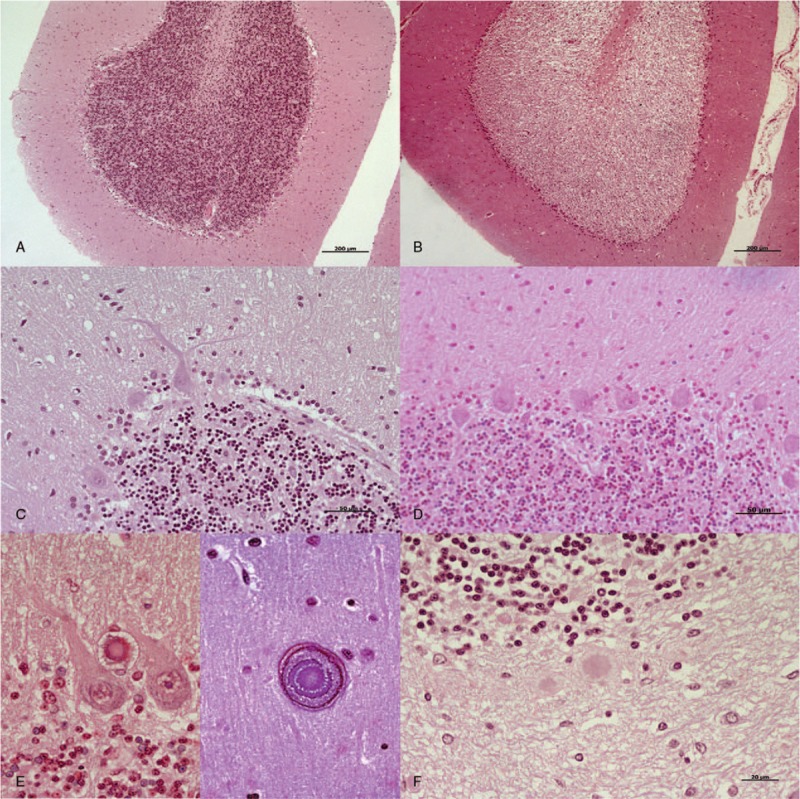
Primary histopathological findings observed by light microscopic examination after HE staining. A, Control samples did not show spongiosis in any cases. B, A total degeneration of the granular layer was found in one control, as illustrated, and in 3 CJD cases. C, An evident decrease in the nuclei count and neurite thickening were frequently observed in Purkinje cells from CJD compared with (D) control cerebella. E, Protein aggregates were clearly evident in the Purkinje cell layer (on the left) and in the molecular layer (on the right) in several prion-affected cerebella. F, Kuru plaques were only found in the cerebellum from 1 CJD patient. Scale bars: 200 μm in (A) and (B); 50 μm in (C) and (D); 20 μm in (E) and (F). CJD = Creutzfeldt–Jakob disease, HE = hematoxylin-eosin staining.

In CJD cerebella, an evident decrease in nuclei count, as well as neurite thickening, was frequently observed in Purkinje cells. Furthermore, protein aggregates were clearly evident in the molecular and Purkinje cell layers by optical microscopic examination. Kuru plaques were exclusively found in 1 CJD patient (Fig. 4).

**Figure 5:**
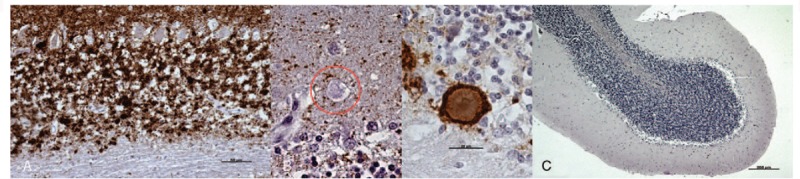
Primary observations provided by light microscopic examination after the application of the IHC protocol for PrPsc detection (brown; hematoxylin counterstaining, blue). A, Purkinje cell layer and white matter from CJD cerebella always corresponded to the lowest intensity of protein deposit immunolabeling. B, Protein aggregates were not always associated with PrPsc immunostaining. C, PrPsc deposits were never observed in healthy controls. Scale bars: 50 μm in (A); 20 μm in (B); 200 μm in (C). CJD = Creutzfeldt–Jakob disease, IHC = immunohistochemistry, PrPsc = pathological prion protein, RT = room temperature.

A wide range of PrPsc deposit profiles was found in the cerebella from CJD patients, although granular and coalescent patterns were the most frequently observed patterns in the molecular and granular layers, respectively. The lowest intensity of protein deposit immunolabeling was detected in the Purkinje cell layer and in white matter. Specific immunostaining against PrPsc was not always present in protein aggregates. No PrPsc deposits were found in any control samples (Fig. 5).

Samples from CJD patients showed an increase in GFAP immunostaining compared with healthy controls, which was especially evident in white matter. Specific astroglial immunostaining always appeared to be associated with protein aggregates, regardless of the presence of PrPsc deposition.

**Figure 6:**
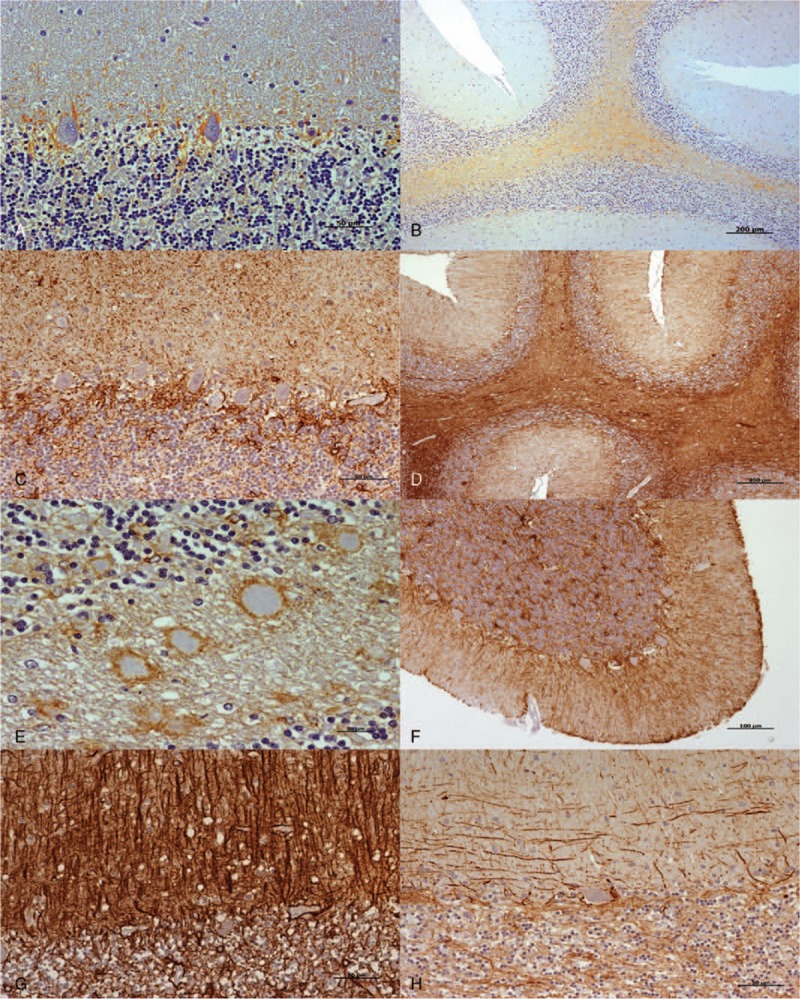
Primary observations provided by light microscopic examination after the application of the IHC protocol for GFAP detection (brown; hematoxylin counterstaining, blue). In comparison with those from healthy controls (A) and (B), the increase in GFAP immunostaining in CJD cerebella was specially evident around Purkinje cells (C) and (D) white matter. E, Protein aggregates always appeared associated with astroglial immunostaining. F, GFAP immunolabeling was evident next to the meninges in some CJD samples. G, An intense radial gliosis in the molecular layers was primarily found in CJD cerebella with higher GFAP intensity. H, Meanwhile, a large number of varicose fibers parallel to the pial surface were observed in CJD cerebella with lower GFAP intensity. Scale bars: 50 μm in (A), (C), (G), and (H); 200 μm in (B) and (D); 20 μm in (E); 100 μm in (F). CJD = Creutzfeldt–Jakob disease, GFAP = glial fibrillary acidic protein, IHC = immunohistochemistry.

Our previous findings regarding astroglial immunostaining in CJD samples^[10]^ were also confirmed here. An increase in the level of immunolabeling surrounding Purkinje cells and next to meninges was observed, and intense radial gliosis or large numbers of varicose fibers parallel to the pial surface were observed in the molecular layer from cerebella with higher or lower GFAP intensity, respectively (Fig. 6).

**Figure 7:**
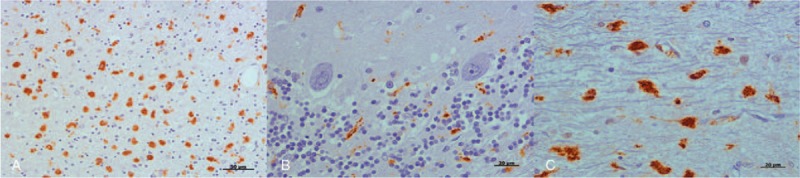
Primary observations provided by light microscopic examination after application of the IHC protocol for reactive microglia detection (brown; hematoxylin counterstaining, blue). A, An amoeboid morphology was found in a degenerated granular layer. B, Purkinje cells were not found to be in close association with reactive microglia. Ramified morphology was primarily observed in this cell layer. C, Meanwhile, amoeboid shapes were more frequently found in white matter. Scale bars: 50 μm in (A); 20 μm in (B) and (C). IHC = immunohistochemistry.

Immunolabelling corresponding to reactive microglia was found scattered over all examined areas in control cerebella. It primarily presented a ramified morphology, except for in the cerebellum that had a degenerated granular layer, where an amoeboid morphology was found. CJD samples demonstrated clear microglial reactivity, as the intensity of immunolabelling was higher in all affected cases compared with controls. Although the distribution of the microglial population (primarily located in white matter or in the molecular and/or granular layers) varied among CJD-affected cases, Purkinje cells were not found to be in a close relationship with this glial population. Moreover, a ramified morphology was primarily observed in the Purkinje cell layer. Immunostaining with amoeboid shapes was more frequently found in white matter (Fig. 7).

**Figure 8:**
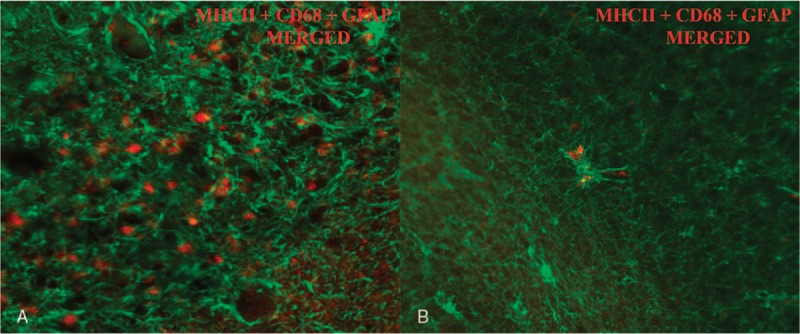
Images obtained by confocal microscopy for the detection of both types of glial cells assessed in this study showed a more extensive interaction between microglia (red) and astroglia (green) in cerebella from (A) CJD patients than in (B) control patients (objectives: ×10, NA-03, capturing ROI and CROP scan). CJD = Creutzfeldt–Jakob disease.

When confocal microscopy was used to determine a possible interaction between the two glial populations studied here, samples corresponding to CJD patients demonstrated interactions to a greater extent than control samples. Astrocytes were observed surrounding microglia in some occasions (Fig. 8).

## 4 Discussion

The function of the central nervous system has been long explained by a neurocentric vision, attributing all the primary information processing activities to neuronal cells. In recent years, several studies have challenged this concept, revealing that non-neuronal cell components, particularly glial cells, perform a dynamic range of functions that are essential for the physiology of the brain.^[11,12]^ Thus, there is growing consensus that a more integrated level of neuroglial interactions needs to be considered to have a comprehensive overview of brain functions and dysfunctions. The observations presented here support the relevance of this interaction in the progression of neurodegeneration associated with prion diseases.

Glial activation had been assumed to be merely a response to pathophysiological events. However, recent data from preclinical and clinical studies have established that immune system-mediated actions contribute to and drive neurodegenerative disease pathogenesis. Neuroinflammation has become a current hypothesis for the basis of neurodegeneration.^[13]^

The fact that microglia play multiple roles in prion diseases is well established. This glial population is assumed to play a major role in the pathological changes in these diseases.^[14]^ The present study assessed a number of CJD cases for reactive microglia in the brains of affected patients in comparison with controls. An increase in reactive microglia has only been described in 2 cases in previous studies.^[15,16]^ Based on animal models it has been suggested that microglial activation precedes neuronal loss.^[17,18]^ In addition, an inflammatory response mediated by microglia, astrocytes, and cytokines in these diseases has been proposed to be an early event in the pathogenesis of disease and appears to remain activated for the duration of the disease.^[19]^ These excessive inflammatory mediators could contribute to neuronal damage. Therefore, the results presented here support the idea that the inhibition of microglial over-activation could be a therapeutic strategy for alleviating microglia-mediated neuroinflammation.

Although the microglial response is thought to be more pronounced in the immature brain^[20,21]^ and in earlier stages of neurodegenerative disease,^[22]^ the findings described here demonstrate that the capacity to process and/or maintain this response against specific stimuli remains, despite aging and disease progression.

It is known that activated microglia up-regulate many surface receptors, such as the MHC, or complement receptors, secreting a variety of soluble biologically active factors. The morphofunctional spectrum of microglia is highly regulated and is reflected by various intermediate states of activation. The malleability of phenotype appears to be the result of the very graded manner of glial cell response to changes in the environment.^[23]^ There is an accepted distinction between phagocytosis by ramified and amoeboid microglia during basal conditions and neurodegeneration, respectively.^[24,25]^ However, the high phagocytic potential of unchallenged microglia may be even further enhanced during neurodegeneration,^[26]^ which could explain the observations of both phenotypes in the present study, as the antibodies that have been used here to recognize immunological activity.

The loss of microglial integrity may also contribute to tissue pathology.^[27]^ It is known that transformed microglial cells are indicative of a loss of tissue homeostasis. Phenotypically transformed microglia should consequently raise suspicions that intrinsic tasks are not being performed efficiently. The amoeboid pattern primarily observed in the white matter from CJD samples, in this study, could be explained by a loss of microglial functions and the consequent enhancement of disease conditions. A low accumulation of PrPsc aggregates was found in this layer.

Microglial activation is followed by astrocyte activation. Activated microglia can initiate and maintain astrogliosis. This suggests that the suppression of microglial over-activity might effectively attenuate reactive astrogliosis.^[28]^ Prion disease pathogenesis entails altered astrocyte function, which may be considered an integral part of the neuroinflammatory response. The presence of activated astrocytes around PrPsc deposits is well documented in prion diseases.^[29]^ In agreement with the observations presented here, glial disorders should not be considered a late reaction to neuronal injury but rather as an intrinsic component of the pathological process. In fact, astrocytes not only form a glial scar in central nervous system diseases,^[30]^ but their activation may occur early in the course of neurodegenerative pathogenesis. Because hypertrophy shown by this glial population eventually results in deficient glutamatergic transmission, they could possibly contribute to cognitive impairment.^[31]^

With this study, a model of neuronal death in prion disease has emerged, indicating that both microglia and astrocytes can either induce or exacerbate neuronal death induced by the abnormal prion protein isoform. Despite some claims that the neurotoxic effect of PrPsc does not require the effects of glia, gliosis and astrogliosis, appear to be clearly involved in the deleterious mechanism of the toxic prion protein. Perhaps a combination of astroglial and microglial effects could explain the neuroinflammation hypothesis by mediating or exacerbating the toxic effects of protein accumulation in the brain. It had been demonstrated that astrocytes can also act on microglia, creating a paracrine, and autocrine feedback loop, whereby microglia and astroglial derived factors regulate each other.^[32,33]^ As a consequence, gliosis would mediate neuronal death. Microglial impairment might paradoxically be sustained by inflammatory cytokines. This suggests that neuropathology could be accelerated through a negative feedback loop. Ultimately, prolonged microglia impairment would also be accompanied by a loss of trophic functions and the elimination of protective properties. In vivo Alzheimer disease experiments have suggested that the coexistence of microglia and astroglia diminishes the ability of microglia to digest and degrade plaques.^[34]^ Therefore, activated astrocytes may exert a regulatory effect (negative feedback) on the phagocytic activity of microglia. The close relationship, demonstrated here by confocal studies, between astrocytes and microglia, combined with the increase in both activated glial populations, would support this cooperative action for neuronal damage.

In addition to microglia and astrocytes, other CNS resident cells, such as endothelial cells, can contribute to neuroinflammation. Endothelial cells, a key element of the neurovascular unit, have been demonstrated to contribute to the entry of misfolded proteins into the brain in prion^[5]^ and Alzheimer disease^[35]^ models. In this model, they have also been demonstrated to influence inflammation, due to mechanisms influencing the efflux of amyloid and neurotoxic species. Additionally, other proteins, such as growth factors and adhesion molecules, may enable the influx of immune cells into the brain.^[36,37]^

In 2004, Marín-Teva et al^[38]^ demonstrated that microglia promoted the death of Purkinje cells in the developing mouse cerebellum. However, the limited detection of reactive microglia and, by contrast, the increased presence of astroglia in the Purkinje cell layer detected in this and previous^[10]^ studies, would suggest the relevant contribution or involvement of astroglia in this task.

With regards to this neuronal cell type, Purkinje cells are the dominant elements involved in the processing of cerebellar information.^[39]^ The cerebellum controls motor coordination, balance, muscle tone, motor learning, and cognition. These functions are partly mediated by neurons in the cerebellar cortex, which receive excitatory input from the somatosensory system and the cerebral cortex. These excitatory inputs are relayed by glutamatergic mossy fibers, originating in the brain stem and spinal cord. A single mossy fiber makes synaptic connections with hundreds of granule cells, and thousands of these cells provide excitatory input to Purkinje neurons. Inhibitory inputs are provided by the molecular layer interneurons and perhaps by Purkinje cells. In agreement with the histopathological findings observed here, the number of Purkinje cells in older animals has been shown to decrease throughout the cerebellar cortex,^[40]^ and Purkinje cells in older animals have been demonstrated to exhibit torpedoes.^[41]^ Moreover, defects have been described as secondary to Purkinje cell loss, including the significant loss of granule cells.^[42]^ It is difficult to determine whether Purkinje neuronal cell loss occurs in a random or orderly fashion, because cerebellar cell death is much more topographically complex that generally appreciated.^[7]^ All these findings about Purkinje cells were similarly described in previous studies on Scrapie (prototype of prion disease), where these neurons were concluded to play a relevant role in neurodegeneration and were simultaneously the most damaged and protected cell type in the studied area.^[4,6]^

In these previous morphological studies on Scrapie, a similar intense radial gliosis was observed as was observed in some of the CJD samples here, which corresponded to terminal stages of the disease. Meanwhile, those showing an increasing number of fibers parallel to the pial surface resembled nonterminal cases (a lesser progression of neurodegeneration). A similar astroglial pattern was also recently described in the cerebella of patients with Alzheimer disease.^[43]^ Some authors point to specific novel glial cells as being good candidates for involvement in the pathogenesis of this neurodegenerative disease,^[44]^ based on their proliferative properties and the capacity for neuron and astrocyte regeneration.^[45]^ Both features result in increased interest in this cell type during neurodegeneration.

Understanding how glial response is triggered in association with protein accumulation under the pathological conditions characteristic of CJD may highlight novel targets for therapeutic intervention, not only for prion disease but also for prion-like disorders. In fact, several attempts to achieve therapeutic interventions have been recently published. One of these studies strongly supports the important contribution of microglia within the prion disease process, identifying the nature of the response through the gene expression analysis of isolated microglia.^[46]^ Our previous studies point to astroglial cells being involved in the pathogenesis of not only CJD but also of Alzheimer disease.^[10]^ The consistent relationship between both microglia and astroglia and protein aggregates, demonstrated in this morphological study, constitutes an innovative contribution to understanding prion diseases. This relationship has also been suggested to be involved in various neurodegenerative disorders, such as Alzheimer's, Parkinson's, or Huntington's diseases, amyotrophic lateral sclerosis, and multiple sclerosis.^[47]^

Finally, it would be relevant to emphasize the importance of studying human brain material to provide reliable results. It has become a common practice in Neurobiology, and in research in general, to extrapolate from experimental models to natural disorders. However, there are large differences between animal models and humans, and this is the likely reason why many conclusions drawn from studies performed on animal models fail to reflect actual mechanisms of disease. Unfortunately, these studies have certain limitations, which prevent the formation of broader conclusions. On many occasions, only partial data on patients are available, and the clinical history, dominant symptoms and even PrP genotype, are unknown.

## 5 Conclusions

Proteinopathies, such as Alzheimer's, Parkinson's, or Huntington's diseases, in which aberrant proteins spread throughout the brain during disease progression, may share a molecular basis and mechanisms of propagation. Therefore, studies elucidating the interaction between gliosis and prion propagation are relevant to these other neurodegenerative diseases, by providing new targets for therapeutic intervention.

The observations presented here demonstrate the involvement of astroglial and microglial cells in all of the CJD cases studied and would support the current controversy about neuroinflammation as a cause of neurodegeneration.

## Acknowledgments

The authors are grateful to Silvia Ruiz and Sonia Gómez for their excellent technical support.

They also thank Prof. Ferrer for technical assistance and advice.

All samples were provided by the human tissue bank from Hospital de Alcorcón (HUFA Biobank, Spanish National Biobank Network). The authors are grateful for this sample supply.

## Author contributions

**Funding acquisition:** Juan Jose Badiola.

**Investigation:** Marta Monzón.

**Methodology:** Rodrigo Salomon Hernández, Moisés Garcés.

**Resources:** Marta Monzón.

**Supervision:** Marta Monzón, Juan Jose Badiola.

**Validation:** Rocío Sarasa.

**Visualization:** Marta Monzón, Moisés Garcés, Rocío Sarasa.

**Writing – original draft:** Marta Monzón.

**Writing – review & editing:** Marta Monzón.
